# Direct Mucosal-Side Fibrosis Cutting for Salvage Endoscopic Submucosal Dissection of Secondary Barrett's Neoplasia Adjacent Multiband Resection Scars

**DOI:** 10.1159/000524269

**Published:** 2022-04-28

**Authors:** Vincent Zimmer, Bert Bier

**Affiliations:** ^a^Department of Medicine, Marienhausklinik St. Josef Kohlhof, Neunkirchen, Germany; ^b^Department of Medicine II, Saarland University Medical Center, Saarland University Homburg, Homburg, Germany; ^c^Institute of Pathology Saarbrücken-Rastpfuhl, Saarbrücken, Germany

**Keywords:** Barrett´s esophagus, Early cancer, Endoscopic submucosal dissection, Perforation, Fibrosis, Multiband resection, Esófago de Barrett, Neoplasia precoce, Disseção endoscópica da submucosa, Perfuração, Fibrose, Ressecção multibandas

A 54-year-old male patient with long-standing Barrett's esophagus underwent multiband ligation endoscopic mucosal resection (MBL-EMR) 1 year previously due to low-risk early cancer (pT1m2, L0, V0, G2, R0). Of note, a nodular-type small Barrett's neoplasia was resected en bloc in one EMR specimen, while the remaining specimens contained areas of low-grade dysplasia without circumscribed lesions. Radiofrequency ablation of the remaining non-dysplastic Barrett's mucosa with preserved acetic acid whitening was scheduled; however, the patient missed several follow-up appointments. At repeat EGD, a secondary Paris 0-IIa lesion estimated at 15 mm and representing a second Barrett's neoplasia emerged adjacent to MBL-EMR scars at oral (towards the mouth) and anterior (towards the sternum) aspects (Fig. 1a, linked color imaging). Acetic acid staining was only abrogated within the lesion itself and endoscopic biopsies confirmed well-differentiated adenocarcinoma. The patient presented for endoscopic submucosal dissection (ESD) after adequate counselling, including alternative surgery. First, an uncomplicated C-shaped incision from the anal side around the posterior (towards the back, or towards 6 o'clock) parts was performed. Unlike the conventional ESD approach to high-grade fibrosis (distant mucosal incision, submucosal approach to fibrosis with or without tunnel technique), direct cutting into the scar area was tried using an articulating ESD knife (3.5-mm ClutchCutter, Fuji, Düsseldorf, Germany). An initial injection of indigo carmine-saline mixture likewise failed to reasonably lift the mucosa. Special attention was paid to first cut in an ultra-superficial fashion as indicated by a crepe paper-like appearance (electrosurgical settings as for mucosal incision: endo cut 1, effect 2, duration 4, interval 1; hemostasis: soft coagulation, effect 4, 100 W; Fig. 1b). Of note, a hard and longer Inoue-type cap was used to adequately grasp the tissue in a superficial fashion. With the incised mucosa continuously pushed aside by the opened scissors, deeper cuts through dense high-grade F2 fibrosis were performed, and this appeared to indicate the correct resection plane (Fig. 1c). The final histopathology confirmed en bloc resection: pT1m2, L0, V0, G1, R0 (Fig. 1d, e).

Salvage ESD for secondary Barrett's cancer after MBL-EMR has rarely been reported and may pose significant challenges due to marked fibrosis [1]. While high-grade fibrosis in ESD is conventionally tackled by distant mucosal incision with a submucosal approach to fibrotic areas, in this approach the fibrotic area is directly approached from the mucosal side by ultra-superficial cuts using an articulating knife followed by pushing aside the fibrotic mucosa followed by deeper cuts into the fibrotic area [2]. While risk of perforation is real, potential benefits may be in the reduction of the overall resection area, thus potentially translating into reduced risk of ESD-related strictures. Albeit such an approach clearly warrants systematic studies in terms of efficacy and safety, as of now the endoscopic treatment in such complex situations equally clearly needs to be individualized [3].

## Statement of Ethics

The patient has given written informed consent for publication (including the publication of images).

## Conflict of Interest Statement

The authors have no conflicts of interest to declare.

## Funding Sources

There was no funding for this work.

## Author Contributions

V.Z. − clinical care, drafting, and finalization of the manuscript; B.B. − pathology care, critical revision, and final approval.

## Figures and Tables

**Fig. 1:**
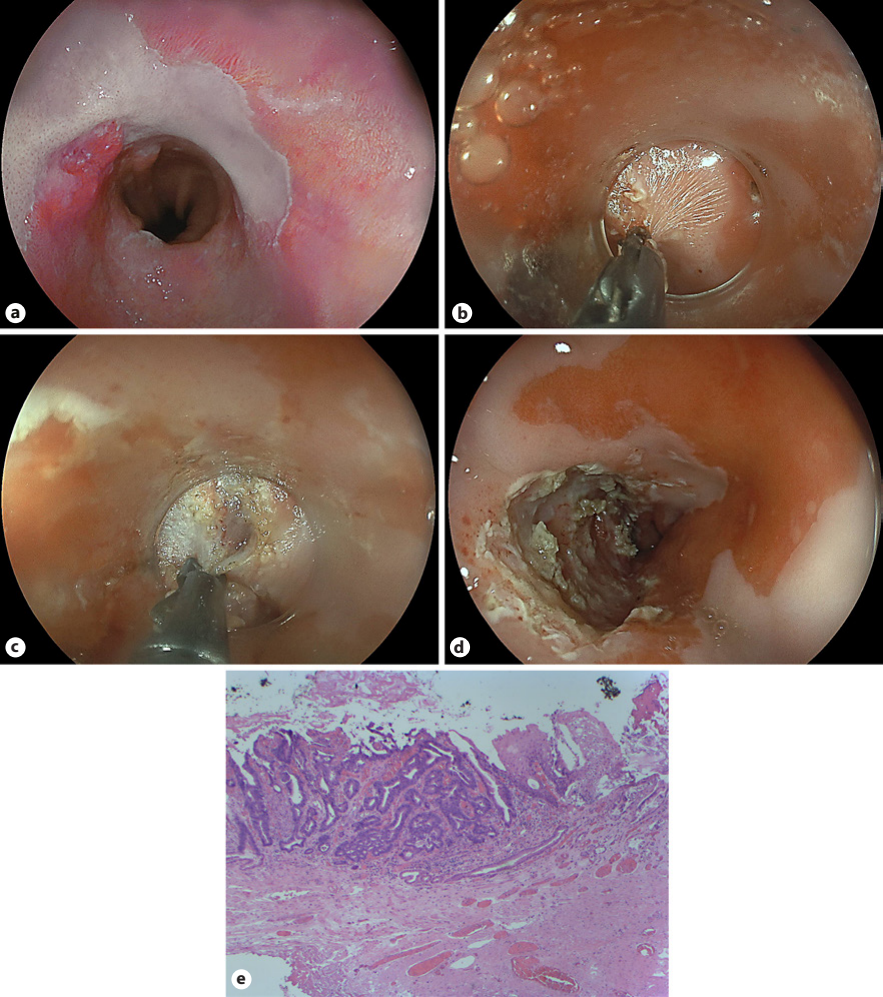
**a** Linked color imaging illustrating an estimated 15-mm, Paris 0-IIa neoplastic lesion adjacent multiband resection scars. **b** Ultra-superficial direct mucosal-side fibrosis cutting using an articulating knife (ClutchCutter, Fuji). **c** Transection of exuberant submucosal F2 fibrosis at later stages. **d** Final operative situs indicating en bloc R0 resection as confirmed by final pathology: pT1m2, L0, V0, G1, R0. **e** Representative histopathology of the Barrett carcinoma. HE stain, ×10.
